# Functional and technical outcomes in acute ischemic stroke patients with hyperdense middle cerebral artery sign treated with endovascular thrombectomy

**DOI:** 10.3389/fneur.2023.1150058

**Published:** 2023-05-25

**Authors:** Yimin Chen, Francesco Diana, Mohammad Mofatteh, Sijie Zhou, Juanmei Chen, Zhou Huang, Weijuan Wu, Yajie Yang, Zhiyi Zeng, Weijian Zhang, Ziqi Ouyang, Thanh N. Nguyen, Shuiquan Yang, José Fidel Baizabal-Carvallo, Xuxing Liao

**Affiliations:** ^1^Department of Neurology and Advanced National Stroke Center, Foshan Sanshui District People's Hospital, Foshan, Guangdong, China; ^2^Department of Neuroradiology, University Hospital San Giovanni di Dio e Ruggi d’Aragona, Salerno, Italy; ^3^School of Medicine, Dentistry and Biomedical Sciences, Queen's University Belfast, Belfast, United Kingdom; ^4^Department of Surgery of Cerebrovascular Diseases, First People's Hospital of Foshan, Foshan, Guangdong, China; ^5^The Second Clinical College, Guangzhou Medical University, Guangzhou, China; ^6^Department of Radiology, Foshan Sanshui District People's Hospital, Foshan, Guangdong, China; ^7^The First School of Clinical Medicine, Southern Medical University, Foshan, China; ^8^Department of Scientific Research and Education, Foshan Sanshui District People's Hospital, Foshan, Guangdong, China; ^9^Department of Neurosurgery, Advanced National Stroke Center, Foshan Sanshui District People's Hospital, Foshan, Guangdong, China; ^10^Department of Neurology and Radiology, Boston University Chobanian & Avedisian School of Medicine, Boston, MA, United States; ^11^Parkinson's Disease Center and Movement Disorders Clinic, Department of Neurology, Baylor College of Medicine, Houston, TX, United States; ^12^Department of Sciences and Engineering, University of Guanajuato, León, Mexico

**Keywords:** endovascular, ischemic stroke, thrombectomy, hyperdense artery, imaging

## Abstract

**Background and objective:**

The hyperdense middle cerebral artery sign (HMCAS) is observed in a proportion of patients with acute ischemic stroke (AIS). This sign reflects the presence of an intravascular thrombus rich in red blood cells. Several studies have demonstrated that HMCAS increases the risk of poor outcomes in AIS patients treated with IV thrombolysis or no reperfusion therapy; however, whether HMCAS predicts a poor outcome in patients treated with endovascular thrombectomy (EVT) is less clear. We aimed to evaluate the functional outcome by the modified Rankin scale (mRS) at 90 days and technical challenges in patients with HMCAS undergoing EVT.

**Methods:**

We studied 143 consecutive AIS patients with middle cerebral artery M1 segment or internal carotid artery + M1 occlusions who underwent EVT.

**Results:**

There were 73 patients (51%) with HMCAS. Patients with HMCAS had a higher frequency of cardioembolic stroke (*p* = 0.038); otherwise, no other baseline difference was observed. No differences in functional outcomes (mRS) at 90  days (*p* = 0.698), unfavorable outcomes (mRS > 2) (*p* = 0.929), frequency of symptomatic intracranial hemorrhage (*p* = 0.924), and mortality (mRS-6) (*p* = 0.736) were observed between patients with and without HMCAS. In patients with HMCAS, EVT procedures were 9  min longer, requiring a higher number of passes (*p* = 0.073); however, optimal recanalization scores (modified thrombolysis in cerebral infarction: 2b-3) were equally achieved by both groups.

**Conclusion:**

Patients with HMCAS treated with EVT do not have a worse outcome at 3  months compared with no-HMCAS patients. Patients with HMCAS required a greater number of thrombus passes and longer procedure times.

## 1. Introduction

Mechanical thrombectomy (MT) has been demonstrated to improve outcomes in select patients with acute ischemic stroke (AIS) secondary to large vessel occlusion (LVO) up to the 24-h time window (1–7). Its efficacy is determined by the size and composition of the occluding thrombus (8–10), the type of occlusion (11–13), and the geometrical features of intracranial arteries (14). Some of this critical information, mainly related to clot composition, is not directly available to the treating physician in a timely manner in usual daily scenarios.

**Figure 1:**
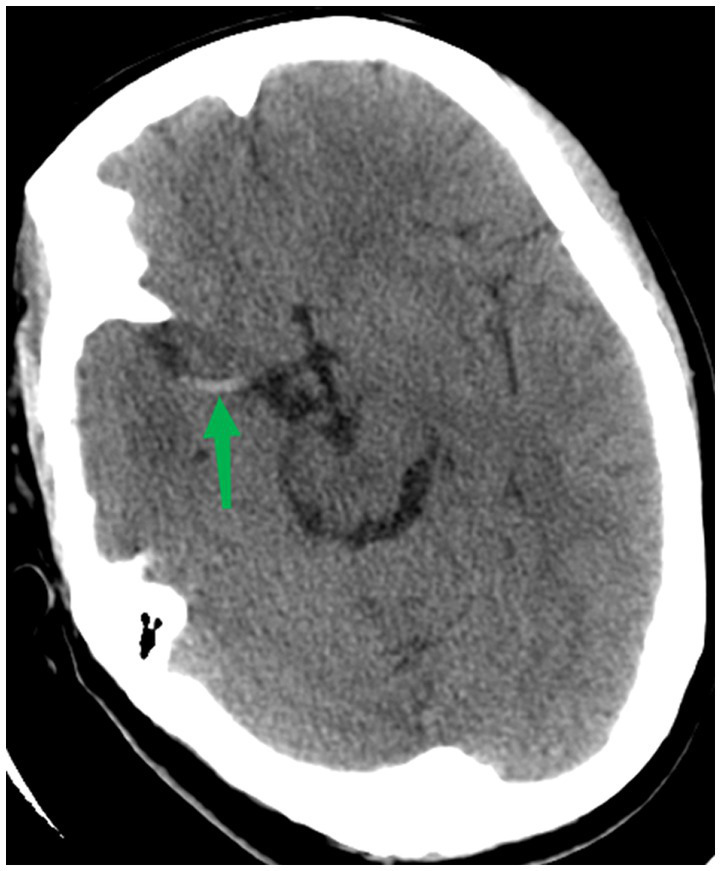
CT Scan showing a typical hyperdense middle cerebral artery sign.

Hyperdense middle cerebral artery sign (HMCAS) is defined as a spontaneous visibility of increased density within the first or second segment of the middle cerebral artery (MCA1 or MCA2) with or without internal carotid artery (ICA) involvement on admission non-contrast CT scan (Figure 1) (8). Several studies have demonstrated that the HMCAS on CT and the blooming artifact on susceptibility-weighted imaging are signs of thrombi rich in red blood cells. Inversely, the absence of HMCAS or blooming artifacts is associated with fibrin-rich thrombi (8). However, differences between HMCAS- and no-HMCAS-related strokes have not been well defined. The aim of this study was to assess the relevance of the HMCAS in patients with AIS treated with endovascular thrombectomy (EVT), comparing the functional and technical outcomes of this cohort with those of patients with no-HMCAS.

## 2. Patients and methods

### 2.1. Study population

We performed a retrospective analysis of prospectively collected thrombectomy databases from two comprehensive stroke centers, Foshan Sanshui District People’s Hospital, between August 2018 and February 2022, and the First People’s Hospital of Foshan, China, between October 2019 and March 2022. Data were derived from the Bigdata Observatory Platform for Stroke in China^1^ and the hospital data platform. We enrolled all consecutive patients with AIS such as those (i) who underwent EVT within 24 h of symptom onset, (ii) ≥ 18 years old, (iii) with the isolated middle cerebral artery (MCA) M1-segment occlusion, or (iv) MCA M1-segment occlusion in a context of a tandem lesion with internal carotid artery (ICA) occlusion. Patients treated with intravenous (IV) tissue-type plasminogen activator (t-PA) were also included in the study. We excluded patients such as those (i) with missing follow-up and (ii) with intracranial occlusions other than the M1 segment. Approximately 80% of cases underwent EVT by using the SOLITAIRE system, and the remaining 20% were treated with previous-generation devices, including MERCI and PENUMBRA.

### 2.2. Study outcomes and assessments

Two vascular neurologists with more than 6 years of practical experience each assessed baseline non-contrast CT scans. HMCAS was determined by visual assessment and by consensus of both experienced stroke neurologists. We collected relevant clinical and demographic data. Clinical features included were age, gender, risk factors, initial premorbid modified Rankin Scale (mRS), National Institute of Health Stroke Scale/Score (NIHSS), and last known normal-to-puncture time (neuroimaging features included: pre-treatment Alberta Stroke Program Early CT Score (ASPECTS)) and occlusion sites. We used the modified thrombolysis in cerebral infarction (mTICI) after thrombectomy to assess the recanalization status (15). An mTICI 2b-3 was defined as successful recanalization. Thrombectomy data included door-to-puncture time, door-to-recanalization time, number of passes, and puncture-to-reperfusion time, and the latter was considered the procedure time.

Patients were followed by trained stroke nurses or vascular neurologists at least twice a month through telephone encounters or in-person consultations. The clinical outcome was assessed by the mRS at 3 months. A favorable outcome was defined as having an mRS of 0–2 while an unfavorable outcome as having an mRS of 3–6. Mortality was defined as an mRS of 6. Symptomatic intracranial hemorrhage (sICH) was defined as any hemorrhage related to transient neurological worsening, manifested by an increase in the NIHSS score of 4 points or higher.

### 2.3. Statistical analysis

Non-normally distributed continuous data were reported as medians along with the interquartile range (IQR). We used the non-parametric Mann–Whitney U-test for non-parametric statistical comparison. Normally distributed variables were reported as means with corresponding standard deviations (SD). We used the student’s *t*-test for parametric comparison. The analysis was performed with IBM SPSS version 26 (IBM-Armonk, NY). We considered a *value of p* of <0.05 as the threshold of statistical significance.

## 3. Results

There were 264 consecutive patients with AIS who underwent EVT. Four patients were lost at follow-up, while among the remaining 260 patients, 148 presented with an M1-segment occlusion, five patients missed the CT scan before EVT, and they were excluded from the study. One hundred and forty-three patients composed the final cohort: 48 female and 95 male subjects, whose mean age was 64.1 ± 14.4 years. At the admission CT scan, 73 patients (51%) presented the HMCAS, while 70 did not (no-HMCAS group). Patients in the no-HMCAS group presented a higher rate of hypertension than those in the HMCAS group (65.71% vs. 49.32%; *p* = 0.047); however, there was no other statistically significant difference in risk factors at baseline between groups (Table 1).

**Table 1:** Comparison of baseline of HMCAS and no-HMCAS.

	HMCAS	No-HMCAS	X^2^/t/z	P
Number	73	70		
Age mean ± SD	64.63 ± 14.19	64.39 ± 12.99	0.287	0.915
Female, *n*, %	22 (30.14)	26 (37.14)	0.787	0.375
Hypertension, *n*, %	36 (49.32)	46 (65.71)	3.929	0.047
Diabetes mellitus, *n*, %	14 (19.18)	13 (18.57)	0.009	0.926
Coronary artery disease, *n*, %	11 (15.07)	9 (12.86)	0.145	0.703
Prior Stroke, *n*, %	10 (13.70)	15 (21.43)	1.480	0.224
Chronic kidney disease, *n*, %	5 (6.85)	8 (11.43)	0.907	0.341
Atrial fibrillation, *n*, %	32 (43.84)	25 (35.71)	0.983	0.321
Smoker, *n*, %	12 (16.44)	16 (22.86)	0.935	0.334
Dyslipidemia, *n*, %	10 (13.70)	9 (12.86)	0.022	0.882
NIHSS Pretreatment (IQR)	15.00 (12.00,18.00)	13.00 (10.75,17.25)	−1.184	0.236
ASPECTS pre-treatment (IQR)	9.00 (8.00,9.00)	8.00 (8.00,9.00)	−0.017	0.986
mRS pre-treatment (IQR)	0.00 (0.00,0.00)	0.00 (0.00,0.00)	−0.713	0.476
Door to needle time (IQR)	44.00 (33.00,53.00)	40.00 (28.00,52.00)	−0.789	0.430
Onset to needle time (IQR)	117.50 (101.50,165.75)	129.00 (104.75,155.75)	−0.257	0.797
Occlusion sites				
Isolated MCA-M1, *n*, %	53 (72.60)	49 (70.00)	0.118	0.731
Tandem (M1+ ICA), *n*, %	20 (27.40)	21 (30.00)		
Mechanism of Stroke (TOAST)				
Large artery atherosclerosis	24 (32.88)	34 (48.57)	3.651	0.056
Cardioembolic	45 (61.64)	31 (44.29)	4.324	0.038
Stroke of other determined etiology	2 (2.74)	3 (4.29)	0.002	0.962
Stroke of undetermined etiology	2 (2.74)	2 (2.86)	0.000	1.000
Technical features				
IV thrombolysis, *n*, %	30 (41.10)	30 (42.86)	0.046	0.831
Last known normal to puncture time (IQR), min	274.00 (201.50,408.50)	308.00 (208.75,490.00)	−0.923	0.356
Door to puncture time (IQR), min	155.00 (119.00,203.50)	146.00 (112.75,190.00)	−0.983	0.325
Door to recanalization time (IQR), min	244.00 (192.50,317.50)	222.00 (168.00,259.25)	−1.882	0.060
Puncture to recanalization time (IQR), min	69.00 (44.50,110.00)	60.50 (35.00,95.50)	−0.935	0.350
mTICI post ≥2b, *n*, %	63 (86.30)	59 (84.29)	0.116	0.734
no of EVT attempts (PASS)	2.00 (1.00,3.00)	1.00 (1.00,3.00)	−1.792	0.073

HMCAS, hyperdense middle cerebral artery sign; mTICI, modified thrombolysis in cerebral infarction; NIHSS, National Institute of Health stroke scale/score.

### 3.1. Etiology and stroke mechanism

According to the TOAST classification, patients with HMCAS presented with a higher rate of cardioembolic stroke (61.64% vs. 44.29%; *p* = 0.038). In contrast, those with no-HMCAS had a trend for a higher frequency of atherosclerosis. One hundred and two patients (71.3%) presented with an isolated M1 occlusion (53 with HMCAS vs. 49 with no-HMCAS), while 41 patients (28.7%) presented with a tandem M1 + ICA occlusion (20 with HMCAS vs. 21 with no-HMCAS).

### 3.2. Technical outcomes

Intravenous t-PA was administered in 60 patients (41.10% of the HMCAS group vs. 34.1% of the no-HMCAS group). There were no differences between groups in terms of door-to-puncture time and last known normal-to-puncture time. The presence of HMCAS negatively affected some technical outcomes. Indeed, the HMCAS was associated with a longer puncture-to-recanalization time (median puncture-to-reperfusion time of 69.00 min vs. 60.50 min, *p* = 0.350) and a higher number of EVT attempts (median of 2.00 vs. 1.00; *p* = 0.073) compared with the no-HMCAS group. However, the proportion of patients achieving successful recanalization (mTICI: 2b-3) was not significantly different between both groups (86.30% in the HMCAS group vs. 84.29% in the no-HMCAS group, *p* = 0.734).

### 3.3. Clinical outcomes

**Figure 2:**
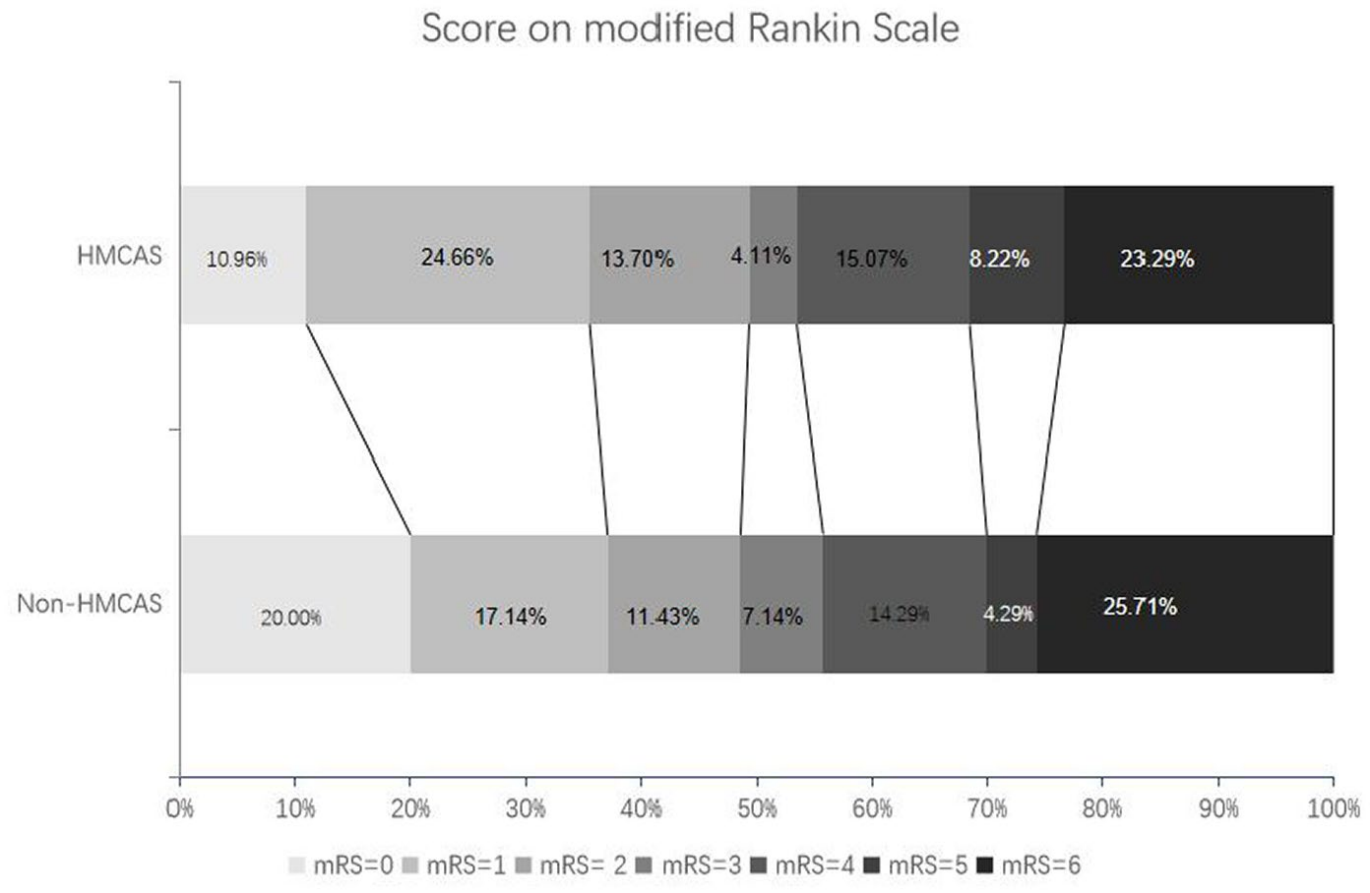
Distribution of modified Rankin Scale scores in patients with HMCAS and non-HMCAS.

Preoperative NIHSS scores (median 15.00 vs. 13.00) and ASPECTS pre-treatment (median 9.00 vs. 8.00) of the two groups were not statistically different. HMCAS did not affect clinical outcomes. Indeed, rates of sICH (12.33% vs. 12.86%; *p* = 0.924), favorable functional outcome (mRS ≤ 2 49.32% vs. 48.57%, *p* = 0.929), and mortality (23.29% vs. 25.71%; *p* = 0.736) were not different between the two groups (Table 2; Figure 2).

**Table 2:** Comparison of outcomes of HMCAS and no-HMCAS.

	HMCAS	No-HMCAS	X^2^/t/z	*p*
sICH, *n*, %	9 (12.33)	9 (12.86)	0.009	0.924
3-month mRS (IQR)	3.00 (1.00, 5.00)	3.00 (1.00, 6.00)	−0.388	0.698
3-month favorable outcome, *n*, %	36 (49.32)	34 (48.57)	0.008	0.929
3-month poor outcome, *n*, %	37 (50.68)	36 (51.43)	0.008	0.929
Mortality at 3 months, *n*, %	17 (23.29)	18 (25.71)	0.114	0.736

Favorable outcome: mRS (0–2) at 3 months. Poor outcome: mRS ≥ 3. sICH, symptomatic intracranial hemorrhage; HMCAS, hyperdense middle cerebral artery sign; IQR, interquartile range; mRS, modified Rankin scale; sICH, symptomatic intracranial hemorrhage.

## 4. Discussion

In the present study, we aimed to assess whether functional and technical outcomes differed between AIS patients with and without the HMCAS treated with EVT. We did not find differences in the distribution of the mRS scores, frequency of sICH, and mortality rates between groups. Although, we found that patients with HMCAS required more thrombus passes during the procedure, leading to longer puncture-to-reperfusion time; however, the recanalization rates did not differ between patients with positive and negative HMCAS.

Several studies have indicated an increased risk for worse functional outcomes at 3 months in AIS patients with HMCAS (16). A recent meta-analysis, including 11 studies with 11,818 patients, showed that those with HMCAS had a 1.56 higher risk of poor outcomes compared with no-HMCAS patients; this effect persisted when considering only patients treated with IV thrombolysis (17). Moreover, the disappearance of HMCAS, which is considered to represent clot dissolution, 22–36 h following IV thrombolysis seems to predict a better functional outcome and survival (18), whereas persistence of the sign is related to the poor outcome at 3 months (19).

However, the adverse predictive value of the HCMAS may be reduced or nullified in patients treated with EVT mainly if the removal of the thrombus is verified, achieving optimal reperfusion. In our study, we did not find differences in functional outcomes at 3 months in patients with and without HMCAS, and both groups achieved optimal recanalization in more than 80% of cases. Kim et al. made a similar observation in 212 patients with HMCAS due to MCA occlusions of segments M1 and M2 treated with EVT, where no differences in outcomes were observed compared to patients with no-HMCAS, irrespective of thrombi location (20). Another study did not show a difference in outcomes at 30 days in patients with positive HMCAS compared with negative HMCAS (21). A meta-analysis including both aforementioned studies showed no significant association of HMCAS with a poor functional outcome at 90 days in patients treated only with EVT: RR: 1.16; 95% CI: 0.99–1.37, *p* = 0.071 (22). The same meta-analysis showed an increased rate of poor functional outcomes in patients with HMCAS undergoing combined EVT, intra-arterial, and IV thrombolysis: RR: 1.40; 95% CI: 1.08–1.80, *p* = 0.010 based on two analyzed studies from the same group of authors (22).

There is evidence that the HMCAS represents a thrombus inside the MCA. However, these thrombi may vary in density, size, and composition; such characteristics may influence recanalization rates (23, 24). Patients with HMCAS may have low recanalization rates following IV thrombolysis owing to high density and other physical–chemical properties of the thrombus, reflected by high Hounsfield units (HU) (25). This has questioned the utility of adding IV thrombolysis to EVT in AIS patients. For example, a meta-analysis enrolling 3,133 patients indicates that outcomes do not differ between patients treated with EVT-only vs. bridging therapy (EVT plus IV thrombolysis) in patients with LVO (26). Additionally, that study showed a lower risk of sICH and clot migration for AIS patients treated with EVT only (26). There is also evidence that HMCAS-negative predicts *in situ* thrombosis owing to atherosclerosis (20). In our study, patients with no-HMCAS had a higher frequency of atherosclerosis as the potential mechanism for the ischemic event (*p* = 0.056), supporting such observation. Moreover, we found that cardioembolic stroke was more frequently observed in patients with HMCAS similar to the study by Kim et al. (20). This supports the notion that red blood cell-rich thrombi underlie HMCAS.

The question of whether a positive HMCAS increases the risk of sICH has been addressed in other studies (17, 27, 28). Although the presence of HCMAS has been associated with acute neurological deterioration following IV thrombolysis, it has not been related to a greater risk of sICH (17, 27, 28). We corroborated such findings in our study as we found no increased risk of sICH in patients with HMCAS.

Our study has limitations. For example, we did not include patients with hyperdense M2 segment of the MCA, owing to few cases encountered during the study period. Moreover, we only enrolled patients from two stroke centers; further studies should include larger series from multiple centers.

In summary, the HMCAS predicts a worse functional outcome in AIS patients treated with IV thrombolysis or no reperfusion therapy. However, such adverse outcomes were lost in our cohort of patients treated with EVT with and without IV thrombolysis. It seems that a more rapid and direct recanalization achieved by optimal EVT eliminates the adverse outcome that is observed in patients with AIS and HMCAS even when other reperfusion techniques, in addition, to EVT are used. No difference in the rate of reperfusion was observed between patients with positive and negative HMCAS following EVT; however, patients with positive HMCAS represented a greater technical challenge owing to a higher number of passes required to achieve recanalization, leading to longer procedure times.

## Data availability statement

The raw data supporting the conclusions of this article will be made available by the authors, without undue reservation.

## Ethics statement

The studies involving human participants were reviewed and approved by Foshan Sanshui District People’s Hospital Review board and First People's Hospital of Foshan Review board. The patients/participants provided their written informed consent to participate in this study.

## Author contributions

YC, FD, XL, and JFB-C drafted the manuscript. MM, TN, SZ, JC, ZH, WW, YY, ZZ, WZ, and ZO analyzed the data and then reviewed and revised the manuscript. JFB-C reviewed and revised the manuscript. All authors reviewed the manuscript and approved the final version of the manuscript.

## Funding

The study was supported by the Foshan Science and Technology Bureau (Grant No. 2220001005022), the Medical Science and Technology Research Foundation of Guangdong Province (Grant No. 20221027164016611), the Foshan 14th Five-Year Plan Key Discipline Foundation, China, the Guangdong provincial TCM Bureau Key Discipline Foundation, China, and the Foshan Competitive Talent Support Project Fund (Brain-Heart Talent Project-Build the Brain-Heart Comorbidity Multi-disciplinary Medical Center).

## Conflict of interest

The authors declare that the research was conducted in the absence of any commercial or financial relationships that could be construed as a potential conflict of interest.

## Publisher’s note

All claims expressed in this article are solely those of the authors and do not necessarily represent those of their affiliated organizations, or those of the publisher, the editors and the reviewers. Any product that may be evaluated in this article, or claim that may be made by its manufacturer, is not guaranteed or endorsed by the publisher.

## Footnotes

^1^https://ss.chinasdc.cn
